# Combined intravenous and intra-articular tranexamic acid administration in total knee arthroplasty for preventing blood loss and hyperfibrinolysis: A randomized controlled trial

**DOI:** 10.1097/MD.0000000000014458

**Published:** 2019-02-15

**Authors:** Yi-Min Zhang, Bo Yang, Xue-Dong Sun, Zhen Zhang

**Affiliations:** Department of Joint Surgery, Weifang People's Hospital, Weifang, P.R. China.

**Keywords:** blood loss, fibrinolytic activity, total knee arthroplasty, tranexamic acid

## Abstract

**Background:**

Total knee arthroplasty (TKA) is a surgical procedure to replace the weight-bearing surfaces of the knee joint to relieve pain and disability. However, blood loss and fibrinolytic activity, accounting for a poor prognosis following TKA operation, were relieved by fibrinolytic inhibitor tranexamic acid (TXA). For a better application of TXA function, we explored the effect of intravenous injection (IV) of TXA combined with intra-articular injection (IA) of TXA in patients after TKA.

**Methods:**

Patients admitted from Weifang People's Hospital from January 2015 to December 2016 who received TKA were injected with 20 mg/kg TXA by IV before TKA (n = 50), 3.0 g TXA by IA after TKA (n = 50), or combination of 20 mg/kg TXA by IV before TKA and 3.0 g TXA by IA after TKA (n = 50). Knee function was assessed using HSS, KSS, NASS, and ROM. In addition, the total blood loss (TBL), hidden blood loss (HBL), maximum hemoglobin (Hb) drop, fibrinolytic activity, as well as incidence of thromboembolism were measured. The patients were followed up for 6 months. The deadline for follow-up was June 2017 and the incidence of thromboembolism events within 6 months after operation was counted.

**Results:**

HSS, KSS, NASS scores, and ROM were elevated after patients receiving TKA. Patients received IV plus IA TXA has decreased TBL, HBL, and maximum Hb drop than those received IV TXA-alone and IA TXA-alone, with reductions in FDP and D-dimer, indicating that IV plus IA TXA injection is superior to prevent blood loss and hyperfibrinolysis during TKA. Age, sex, type of femoral prosthesis, and the injection method of TXA were risk factors for HBL of patients after receiving TKA.

**Conclusions:**

The aforementioned results demonstrate that TKA is an effective surgery, and IV plus IA TXA injection functions more effectively in reducing blood loss and fibrinolytic activity in patients, which is a clinical factor of occult hemorrhage.

## 1 Introduction

Total knee arthroplasty (TKA) is a common and safe surgical procedure used to relieve symptoms of severe knee arthritis.^[1]^ Although TKA has been considered as safe and effective, such factors as age, diabetes, and greater body mass index (BMI) may increase the morbidity after TKA.^[2]^ The deep surgical site infection is a fatal complication after the TKA operation.^[3]^ Furthermore, the extensive blood loss of patients during and after TKA can result in anemia, which accounts for an increased need of transfusion and longer hospitalization, contributing to clinical and economic burden.^[4]^ In addition to blood loss, venous thromboembolism (VTE), including deep vein thrombosis (DVT), and pulmonary embolism (PE) developed in patients underwent TKA, is another life-threatening complication.^[5,6]^ As has previously reported, fibrinolytic activity is associated with VTE.^[7]^ To reduce blood loss of patients after TKA operation, postoperative leg position has been reported as a convenient and efficient technique to reduce blood loss and improving the recovery of joint knee.^[8]^ In order to decrease the development of VTE, the non-vitamin K antagonists oral anticoagulants (NOAC), including apixaban, is administered as an option for VTE treatment in a simple and fixed-dose regimen.^[9]^ Recently, tranexamic acid (TXA) has been reported with efficiency in anti-liver injury and fibrin clots in a mouse model of chronic bile duct injury.^[10]^ Also, TXA has been widely performed for patients after TKA to reduce blood loss and inhibit fibrinolytic activity by intravenous injection and oral taking.^[11]^ Thus, this study was conducted to assess the use of TXA in patients undergoing TKA.

TXA is a synthetic derivation of amino acid lysine, which is a type of antifibrinolytic agent that inhibits the fibrin clot dissolution by blocking the interaction of plasmin with fibrin and binding to plasminogen.^[12]^ As has been mentioned above, oral taking and intravenous injection are the 2 major ways for TXA administration after TKA operation. Apart from those means, topical application of TXA to the bleeding wound surface decreases blood loss in patients without systemic complication following major surgeries.^[13]^ A previous research demonstrated the intravenous injection of TXA (IV TXA) as a more effective way to reduce transfused units and hemoglobin drop than TXA injection by drain, which functioned more effectively in postoperative drainage reduction.^[14]^ Besides, intra-articular injection of TXA (IA TXA) was found to be functional both in reducing postoperative blood loss and ameliorating knee joint swelling of patients undergoing TKA.^[15]^ For the consequences resulted from blood loss and fibrinolytic activity after TKA, this study was performed to analyze a better utilization of TXA by comparison of 3 different injection strategies, IV TXA-alone, IA TXA-alone, and IV plus IA TXA, as to benefit TKA therapy.

## 2 Materials and methods

### 2.1 Ethics statement

The protocol of this study was approved by the Ethics Committee of Weifang People's Hospital. Written informed consents were obtained from all patients or their parents or guardians.

### 2.2 Study subjects

From January 2015 to December 2016, 150 patients aged 40 to 80 years old underwent TKA initially were included in our study. Patients all met the following criteria: patients were treated with supplemental blood volume ≤2000 mL within 20 hours following TKA; patients had normal platelet amount and coagulation function before TKA operation; TKA operation and the postoperative nursing were performed by the same group of doctors and nurses; the hospital for special surgery knee score (HSS) of knee joints before TKA operation <60; patients had no abnormality in the venous system of the lower limbs with Color Doppler ultrasonography before TKA operation. Exclusion criteria including: before TKA operation, patients are in need of antibiotic treatment for their pulmonary infection or urinary tract infection; patients had contraindication to TKA; patients at a high risk of developing thrombosis (such as patients who had received stent implantation and who carrying a heart pacemaker); patients suffered from malignant tumors. According to patient's informed consent and the random number table, all enrolled patients were classified into IV TXA-alone group, IA TXA-alone group, and IV plus IA TXA group, with 50 cases in each group. They received either drugs or control solutions in the surgical joints with the same disposable syringe. The syringe was labeled with numbering code, which was used to distribute concealment and blindness to ensure the randomness of the operation process by pharmacists independently.

### 2.3 Perioperative management

Before the operation, all patients received systematic examination of routine blood test, coagulation function, electrocardiogram, and knee joint x-ray. On the day before TKA, they were guided to perform a proper set of quadriceps contractions and dorsal extension exercise of ankle joint, as well as to receive antibiotic treatment. The TKA operation was carried out based on the aforementioned procedures: primarily, patients received epidural anesthesia in a supine position. Next, TKA was carried out using strict hemostasis (when systolic blood pressure was >150 mmHg). Then, an incision was made at the center of the joint knee; a medial parapatellar approach was performed that skin and subcutaneous layers were incised successively along the medial patellar, and the fat pad, internal and external meniscus under which were removed. After adjustment of soft tissue balance, the reset and adjustment of thighbone prosthesis and tibial prosthesis was performed using the cemented posterior-stabilized prosthesis (Biomet Inc., Warsaw, IN). For active hemorrhage, ligation or electric coagulation was used to stop hemorrhage. For extensive bleeding of wounds and osteotomy, gauze packing was used to compress and stop bleeding until the bleeding was controlled during operation.^[16,17]^ The incision was fully washed before it was sutured. One drainage tube was placed in the articular cavity and the incision was closed. After 24 hours, the drainage tube was removed and the wound was dressed under pressure. The epidural anesthesia tube was removed immediately after the operation. After TKA operation, all patients received the same analgesic treatment and a routine antibiotic treatment to avoid infection for 2 to 3 hours. Twelve hours after the operation, patients were continuously given 10 mg rivaroxaban (1 time/d) for 2 weeks.

### 2.4 TXA treatment

Patients in the IV plus IA TXA group underwent intravenous injection of 20 mg/kg TXA before the incision, who also received articular injection of 3.0 g TXA after it was sutured. Patients in the IV TXA-alone group had intravenous injection of 20 mg/kg TXA before the incision, and those in the IA TXA-alone group received articular injection of 3.0 g TXA after it was sutured.

### 2.5 Evaluation of knee joint function, blood loss, and postoperative events

After the operation, the HSS scores of patients, including pain, function, range of motion (ROM), muscular strength, knee flexion deformity, and stability, were measured and recorded, with higher score suggesting better knee joint function. Keen Society Score (KSS) contains the knee score (pain, stability, and ROM) and the function score (walking distance and stair climbing), and higher KSS score indicates better knee joint function.^[18,19]^ According to the North American Spinal Association (NASS),^[20]^ patients should answer the following questions, including does the operation meet patients’ expectations? How do patients evaluate the overall therapeutic effect? From the 8 components of Short-Form-36 (SF-36), the average physical component summary (PCS), and average mental component summary (MCS) of patients were evaluated. PCS included physical function, physical pain, and general health components of SF-36, while MCS included vitality, social function, and mental health components of SF-36. ROM, the maximum active and passive ROM of joint knee, was also applied for function evaluation. Besides, total blood loss (TBL), hidden blood loss (HBL), and maximum hemoglobin (Hb) were recorded and estimated, among which TBL was calculated by Gross formula^[21]^ and HBL = TBL – visible blood loss (VBL).^[22]^ TBL after surgery is calculated by subtracting the fluid in the aspirator from the flushing fluid used during the operation, plus the weight gain of the hemostatic cloth. HBL is the total amount of blood loss after operation minus the visible amount of blood loss after operation. VBL after operation includes blood transfused 6 hours after operation and blood loss infiltrated into dressing after operation. Thromboembolism of patients, such as idiopathic venous thromboembolism (IVT), DVT, and PE were observed 6 months after the operation to analyze the incidence of thromboembolism. Also, at the day before operation (t1), 1 day after operation (t2), and 3 days after operation (t3), fasting venous blood was collected from patients so as to assess fibrinolysis, including fibrin degradation products (FDP) and D-dimer.

### 2.6 Follow-up

Post-discharge follow-up was carried out for all patients for 6 months, till June 2017, which was conducted via telephone interview or in-office visit. During the follow-up, imaging examination was required, and lower limbs were photographed in standing position, together with radiograph of anteroposterior and lateral knee joint. No infection occurred after 6 months of follow-up.

### 2.7 Statistical analysis

All data were analyzed using SPSS 21.0 software (IBM, Armonk, NY). Measurement data were expressed as mean ± standard deviation. The *t* test was used for comparison between 2 groups and one-way analysis of variance (ANOVA) was for comparison among multiple groups. And repeated data were analyzed by repeated measures ANOVA. Enumeration data were presented as (case [%]), and analyzed by chi-square test. The univariate analysis was used for the relationship between HBL and clinical indicators for patients after TKA. The stepwise multivariate linear regression analysis was carried out to reassess the risk factors affecting HBL for patients after TKA. *P* <.05 suggested a significant difference.

## 3 Results

### 3.1 Clinicopathological characteristics among patients in the IV TXA-alone, IA TXA-alone, and IV plus IA TXA groups

Primarily, we analyzed the clinicopathological characteristics of patients in the IV TXA-alone, IA TXA-alone, and IV plus IA TXA groups. There were 12 men and 38 women in the IV TXA-alone group, with a mean age of (63.12 ± 8.79) years old, and a mean BMI of (27.16 ± 2.43) kg/m^2^. Among them, there were 21 cases with valgus deformity, and 29 cases with varus deformity. In the IA TXA-alone group, there were 10 men and 40 women who had a mean age of (59.86 ± 12.01) years old, and a mean BMI of (25.04 ± 4.28) kg/m^2^. In this group, 19 cases with valgus deformity, and 31 cases with varus deformity were observed. And in the IV plus IA TXA group, there were 16 men and 34 women with a mean age of (63.30 ± 12.95) and a mean BMI of (23.84 ± 4.79) kg/m^2^. A total of 23 cases with valgus deformity and 27 cases with varus deformity were recorded. By comparison of clinicopathological characteristics among the 3 groups, it can be concluded that no significant difference was observed in terms of age, sex, distribution of knee injury, disease, and deformity type (all *P* > .05) (Table 1).

**Table 1:**
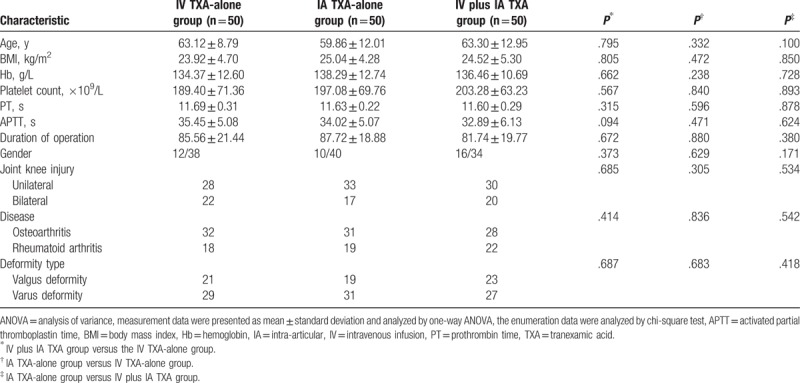
Clinicopathological characteristics of patients among the IV TXA-alone, IA TXA-alone, and IV plus IA TXA groups.

### 3.2 Joint knee function is improved after TKA operation

Then, the HSS score, KSS score (knee score and function score), NASS score, and ROM of patients were assessed before and after TKA operation by which to evaluate its clinical efficacy. After TKA operation, all 150 patients had elevated HSS, KSS, and ROM compared with those before the operation (all *P* < .05) (Table 2). The increased HSS score, KSS score, and ROM indicated that the joint knee function was improved after TKA operation.

**Table 2:**
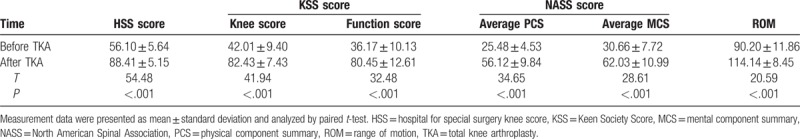
HSS score, KSS score, NASS score, and ROM were increased after TKA operation (n = 150).

### 3.3 Therapeutic efficacy of IV TXA-alone, IA TXA-alone, and IV plus IA TXA following TKA operation

Therapeutic efficacy of TKA combined treatment with IV TXA-alone, IA TXA-alone, and IV plus IA TXA was evaluated by comparing the HSS, KSS (clinical score and functional score), and ROM scores of patients among 3 groups. The results (Table 3) revealed that there were no significantly different HSS score, KSS score, NASS score, and ROM among the 3 groups (all *P* > .05).

**Table 3:**
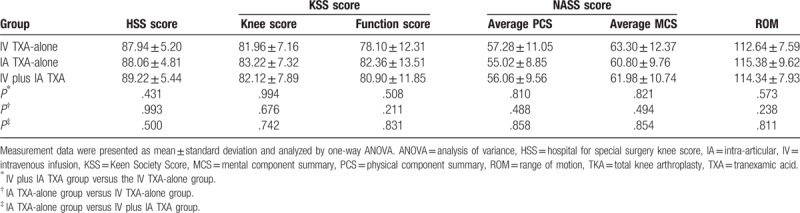
HSS score, KSS score, and ROM among patients in the IV TXA-alone, IA TXA-alone, and IV plus IA TXA groups.

### 3.4 IV plus IA TXA contributes to less blood loss and lower Hb following TKA operation

**Figure 1:**
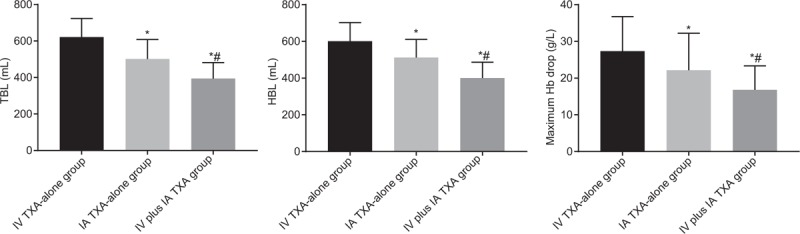
TBL, HBL, and maximum Hb drop were decreased in patients with treatment of IV plus IA TXA following TKA operation. Measurement data were presented as mean ± standard deviation and analyzed by one-way ANOVA, n = 50; ^∗^, *P* < .05 versus the IV TXA-alone group; ^#^, *P* < .05 versus the IA TXA-alone group. HBL = hidden blood loss, Hb = hemoglobin, IA = intra-articular, IV = intravenous infusion, TBL = total blood loss, TXA = tranexamic acid.

TBL, HBL, and maximum Hb drop were analyzed to assess the efficacy of the combination of TKA operation with IV TXA-alone, IA TXA-alone, and IV plus IA TXA. As shown in Table 4 and Fig. 1, the TBL, HBL, and maximum Hb drop were lower in the IV plus IA TXA group than those in the IV TXA-alone and IA TXA-alone groups (all *P* < .05) and the TBL, HBL, and maximum Hb drop were higher in the IV TXA-alone group than those in the IA TXA-alone group (all *P* < .05). The above results demonstrated that blood loss of patients was reduced after receiving TKA operation combined with IV plus IA TXA treatment.

**Table 4:**
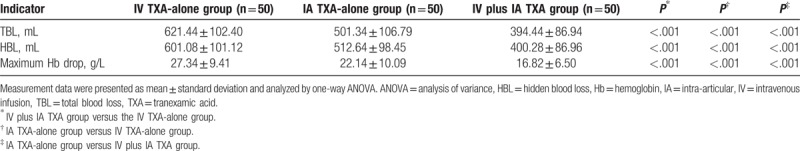
TBL, HBL, and maximum Hb drop were decreased patients in the IV plus IA TXA group than in the IV TXA-alone and IA TXA-alone groups after TKA operation.

### 3.5 IV plus IA TXA reduced fibrinolytic activity following TKA operation

**Figure 2:**
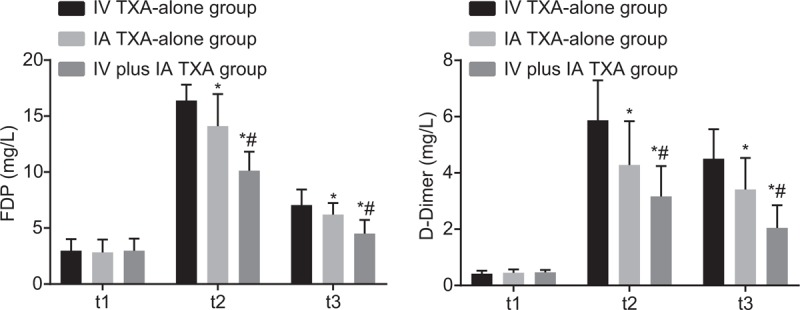
FDP and D-dimer were decreased in the IV plus IA TXA group than those in the IV TXA-alone and IA TXA-alone groups 1 day and 3 days after TKA. Measurement data were presented as mean ± standard deviation and analyzed by repeated measures ANOVA, n = 50; ^∗^, *P* < .05 versus the IV TXA-alone group; ^#^, *P* < .05 versus the IA TXA-alone group. FDP = fibrin degradation products, IA = intra-articular, IV = intravenous infusion, TXA = tranexamic acid.

FDP and D-dimer were indicators of fibrinolysis, so we measured the FDP and D-dimer in patients after receiving TKA operation combined with IV TXA-alone, IA TXA-alone, and IV plus IA TXA. After repeated measurement of FDP and D-dimer 1 day before, 1 day, and 3 days after the TKA operation, the results showed that the changes of FDP and D-dimer between 2 groups and along with time were statistically different (*F*[3.394, 249.424] = 54.093, *I* < 0.001; *F*[3.025, 222.347] = 30.020, *P* < .001) and were time-dependent (*F*[1.697, 249.424] = 1967.114, *P* < .001; *F*[1.513, 222.347] = 648.114, *P* < .001). By repeated measures ANOVA, FDP, and D-dimer were not significantly different among the IV TXA-alone, IA TXA-alone, and IV plus IA TXA groups 1 day before TKA operation (all *P* > .05). However, FDP and D-dimer were significantly reduced in the IV plus IA TXA group than those in the IV TXA-alone and IA TXA-alone groups (all *P* < .05), which was obviously increased in the IA TXA-alone group than in the IV TXA-alone group (all *P* < .05) (Table 5 and Fig. 2). These findings indicated less fibrinolysis in patients receiving TKA operation combined with IV plus IA TXA treatment.

**Table 5:**
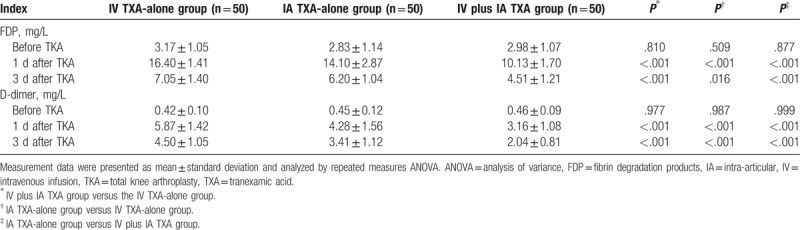
FDP and D-dimer were reduced in patients in the IV plus IA TXA group than in the IV TXA-alone and IA TXA-alone groups after TKA operation.

### 3.6 IV plus IA TXA revealed no increased risk of thromboembolism following TKA operation

VTE occurred in patients after receiving TKA could seriously affect the therapeutic effect of TKA. Therefore, we detected the incidence of thromboembolism in patients from the IV TXA-alone, IA TXA-alone, and IV plus IA TXA groups. The result shown in Table 6 displayed that the incidence of thromboembolism in the IV TXA-alone group was 18.00%, which was 16.00% and 20.00% in the IA TXA-alone group and the IV plus IA TXA group, respectively. Thus, no significantly different incidence was seen among the IV TXA-alone, IA TXA-alone, and IV plus IA TXA groups (*P* > .05).

**Table 6:**
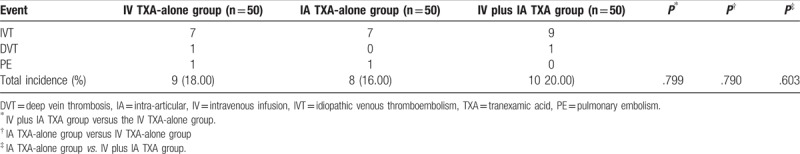
The incidence of thromboembolism among patients in the IV TXA-alone, IA TXA-alone, and IV plus IA TXA groups.

### 3.7 Age, sex, BMI, type of femoral prosthesis, and the injection method of TXA were risk factors for postoperative HBL

Then, we adopted univariate analysis to analyze the factors which may affect the HBL after patients receiving TKA. The results showed that different age, sex, BMI, type of femoral prosthesis, and the injection method of TXA could potentially influence the HBL (all *P* < .05), while preoperative diagnosis, the occurrence of thromboembolism, and deformity type of joint knee were not significantly related with HBL after TKA operation (all *P* > .05) (Table 7).

**Table 7:**
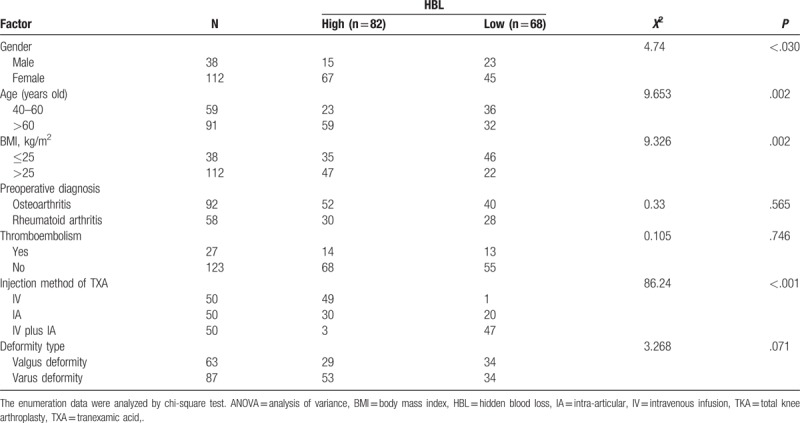
Single risk factors for HBL after TKA operation were analyzed by one-way ANOVA.

The stepwise multivariate linear regression analysis was carried out to assess the risk factors for HBL after TKA operation with HBL as the dependent variable and the risk factors mentioned above (age, sex, BMI, type of femoral prosthesis, and the injection method of TXA) as the independent variables, and the results revealed that age, sex, and the injection method of TXA were risk factors for HBL after TKA operation (Table 8).

**Table 8:**
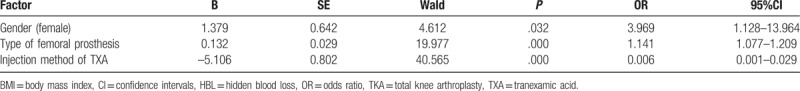
Multivariate risk factors for HBL after TKA operation were analyzed by the stepwise multivariate linear regression analysis.

## 4 Discussion

Despite advancement in TKA for patients with end-stage knee arthritis, only 82% to 89% of them obtained satisfactory outcomes with their primary TKA.^[23]^ Deep infection, stiffness, poor flexion gain, malalignment, unexpected pain, loosening are main reasons leading to the dissatisfaction of patients undergoing TKA.^[24–26]^ Apart from that, the poor prognosis of TKA is mostly caused by the intraoperative pneumatic thigh tourniquet applied during TKA which increases local fibrinolytic activity and contributes to the postoperative blood loss.^[16]^ To solve that problem, the fibrinolysis inhibitor TXA has been used to reduce blood loss and transfusion when it is given to patients intravenously, which can directly target the bleeding site and systematically suppress the occurrence of thromboembolic complications when it is administered to patients by topical and IA application.^[27]^ In this study, we compared the recovery of joint knee after TKA by receiving TXA through different injection strategies, and the results showed that IV plus IA TXA was more effective to prevent blood loss and decrease fibrinolytic activity in patients undergoing TKA than IV TXA-alone and IA TXA-alone.

Primarily, the TBL, HBL, and maximum Hb drop of patients were detected after they received TKA, and it could be seen that patients treated with IV plus IA TXA had decreased TBL, HBL, and maximum Hb drop, indicating that blood loss was slowed down. TXA is an antifibrinolytic agent which helps to reduce blood loss during major operation.^[28]^ TXA in orthopedics has been widely used in TKA to prevent bleeding due to its efficacy and ease of use both in IV and IA.^[29]^ In a recent study, the performance of one bolus of IV TXA in patients following TKA was shown to decrease TBL, HBL, and maximum Hb.^[30]^ In addition to the IV TXA, the combined IA TXA was suggested effective in reducing blood loss in total hip arthroplasty.^[31]^ Moreover, in line with our study, the IV plus IA TXA treatment was revealed to have a 37% reduced blood loss compared with IV TXA-alone at 24 and 48 hours after TKA, where no thromboembolic complications occurred.^[32]^ Additionally, the inhibitory effect of TXA on the fibrinolytic activity was previously demonstrated in several studies.^[33,34]^

Secondly, repeated measures ANOVA was adopted to determine the FDP and D-dimer levels in patients receiving IV TXA-alone, IA TXA-alone, and IV plus IA TXA after TKA. It was shown that FDP and D-dimer levels decreased more in patients after TXA injection, indicating a reduction in fibrinolytic activity. Fibrinolysis, a final division of hemostasis, is essential in maintenance of blood flow and vessel patency against the procoagulant properties; monitoring FDP and D-dimer levels may aid fibrinolysis evaluation.^[35,36]^ According to a previous study, FDP and D-dimer were found to be involved in numerous vascular diseases.^[37]^ The D-dimer level is almost doubled in patients during or after TKA, and the higher D-dimer is generally related to an increased risk of DVT.^[38,39]^ As has been reported, IV TXA-alone could reduce FDP, d-dimer to aid suppression of fibrinolysis without an increasing risk of complications,^[30]^ which is greatly in consistent with our results. Furthermore, our study demonstrated the combination of IV and IA TXA contributed to a reduction in fibrinolysis following TKA operation without an increasing incidence of thromboembolism. In view of the above findings, the inhibitory effect of IV plus IA TXA on fibrinolytic activity can be easily seen.

Thirdly, we analyzed the potential risk factors contributing to the HBL after TKA based on the univariate analysis and stepwise multivariate linear regression analysis. The results showed that age, sex, BMI, type of femoral prosthesis, and the injection method of TXA were influential factors for HBL after TKA. A previous study reported that sex, operation duration time, and TXA use were among the elements that influenced HBL after TKA, and specifically, male patients had a greater blood loss than female patients, and longer operation procedure led to increased HBL.^[40]^ High BMI is associated with increased risk of complication after TKA.^[41]^ Besides, it was indicated by a previous study that a closed-box knee prosthesis could possibly reduce blood loss after TKA.^[42]^ Apart from these factors mentioned in the previous study, the findings in this study further suggested that the injection method of TXA was a risk factor for HBL after TKA. Patients with treatment of IV plus IA TXA had less HBL than those with IV TXA-alone or IA TXA-alone.

## 5 Conclusions

In conclusion, from the findings of our study along with the previous research results in line with ours, it was revealed that the IV plus IV TXA functioned more effectively in reducing blood loss and fibrinolytic activity in patients after receiving TKA. For the lack of theoretical and experimental researches, forthcoming studies should be paid attention to more efficient ways to prevent the complications following TKA, thus providing benefits for patients with severe injured joint knee.

## Acknowledgments

The authors would like to acknowledge the helpful comments on this paper received from our reviewers.

## Author contributions

Yi-Min Zhang designed the study and prepared the figures and tables. Xue-Dong Sun and Bo Yang collated the data, designed and developed the database, carried out data analyses and produced the initial draft of the manuscript. Zhen Zhang contributed to drafting the manuscript. All authors have read and approved the final submitted manuscript.

**Conceptualization:** Yi-Min Zhang, Bo Yang, Xue-Dong Sun.

**Data curation:** Yi-Min Zhang, Bo Yang, Xue-Dong Sun.

**Formal analysis:** Yi-Min Zhang, Bo Yang.

**Investigation:** Yi-Min Zhang.

**Project administration:** Xue-Dong Sun.

**Resources:** Xue-Dong Sun.

**Supervision:** Zhen Zhang.

**Writing – original draft:** Xue-Dong Sun, Zhen Zhang.

**Writing – review & editing:** Zhen Zhang.
