# Combined intravenous and intra-articular tranexamic acid administration in total knee arthroplasty for preventing blood loss and hyperfibrinolysis: A randomized controlled trial: Erratum

**DOI:** 10.1097/MD.0000000000015000

**Published:** 2019-03-22

In the article, “Combined intravenous and intra-articular tranexamic acid administration in total knee arthroplasty for preventing blood loss and hyperfibrinolysis: A randomized controlled trial”,^[1]^ which appears in *Medicine*, Volume 98, Issue 7, the corresponding authors’ emails should be zhangyimin69@sina.com and 116625326@qq.com.

The authors would like to note the following funding information: This study was funded by Weifang Health and Family Planning Commission Scientific Research Project (2017wsjs002).
